# Supplementary material for: Study of the knowledge about gamification of degree in primary education students
Source: PLoS One. 2022 Mar 29;17(3):e0263107. doi: 10.1371/journal.pone.0263107 (PMC8963584; doi:10.1371/journal.pone.0263107)
Supplement: Attachment — Submitted filename: Response to Reviewers.pdf [file pone.0263107.s001.pdf]

Dear reviewers,

First we would like to thank the reviewers who have corrected the manuscript. We would really like to convey our sincere thanks for each, and every suggestion that they made. With them, we have been able to significantly enrich the article sent to the journal. We are very grateful.
Here are all the suggestions made by each of the reviewers that we had included:

Reviewer 1:

- We have changed the designation of our students to "Degree in Primary Education"
- We just changed the introduction phrases, as you had indicated in the corrections
- In reference to the research question, and its subsequent treatment in the manuscript, a series of doubts arise. The following lines attempt to make an evaluative synthesis of this aspect.
    - There are three essential aspects that circumscribe the study problem: knowledge, use and difficulties.
        - Yes, are them
    - In the Methods section, it is reported that the aim is to ascertain "opinions and knowledge".
        - We added in method: Knowledge is measured through students' own self-perception, while opinions about the use of gamification as a resource and integration difficulties are vicarious experiences that have been seen, but have not been personally experienced.
    - In the instrument section, a scale to measure opinion is mentioned.
        - We added to instrument: To answer the questions posed in this study, an ad hoc scale has been used to measure the knowledge of future teachers about gamification as a didactic resource. In order to know the opinion on the use of this resource in the school environment, three indicators have been identified: the possibility of application, the opportunities for use and its level of

integration in the classroom. And, to know the difficulties for their integration in the classroom, they have asked directly about different situations that may lead to problems.

  - The results focus on knowledge. The previous explanation allows to readers to understand the manuscript

- The information about single participant were excluded
- We changed the age from 18 to 21, it was a mistake
- We have unified the term gender
- We added the specialty to the title, as your suggestion
- The information about consent was modified because the term that we used was not the correct one
- The expression “the reasons that the teacher give for their reluctance…” the term teacher was a mistake and we changed for students, because we refereed to the “future teachers”
- As for the table 4 we analyzed their perception. If they considered that they have a low knowledge about gamification but with the information that they received is enough to know that it is an useful didactic strategy
- We changed the place of table 5 to 6 and vice versa
- We also added the U.S.C for U. Santiago de Compostela in the Table 6
- In the conclusions we added information from nowadays to complete aour affirmations

Reviewer 2:

- We added the aim to the manuscript
- The method was completed
- The sample size was also completed
- The contribution of this manuscript it is included in discussion and conclusions

Reviewer 3:

- The literature and also the discussion and conclusions were completed with more references

Finally, in reference to the other comments from the reviewer 2 and 3 we do not answer because the reviewer 1 just did it and we answered there.

Thank you all
