# Supplementary material for: Study of the knowledge about gamification of degree in primary education students
Source: PLoS One. 2022 Mar 29;17(3):e0263107. doi: 10.1371/journal.pone.0263107 (PMC8963584; doi:10.1371/journal.pone.0263107)
Supplement: Attachment — Submitted filename: Response to Reviewers.docx [file pone.0263107.s002.docx]

PONE-D-21-07412R1

Study of the knowledge about gamification of Degree in Primary Education students

PLOS ONE

**Comments to the Author**

1. If the authors have adequately addressed your comments raised in a previous round of review and you feel that this manuscript is now acceptable for publication, you may indicate that here to bypass the “Comments to the Author” section, enter your conflict of interest statement in the “Confidential to Editor” section, and submit your "Accept" recommendation.
The [PLOS Data policy](http://www.plosone.org/static/policies.action#sharing) requires authors to make all data underlying the findings described in their manuscript fully available without restriction, with rare exception (please refer to the Data Availability Statement in the manuscript PDF file). The data should be provided as part of the manuscript or its supporting information, or deposited to a public repository. For example, in addition to summary statistics, the data points behind means, medians and variance measures should be available. If there are restrictions on publicly sharing data—e.g. participant privacy or use of data from a third party—those must be specified.

Reviewer #3: I would like to congratulate the authors for the work they have carried out concerning the revision of the paper. In my opinion, the paper is now strengthened, specially concerning the Methodology section. However, some of my previous revision comments are not addressed in the revised version on the paper, nor in the response to reviewers.

Comments about literature review:

Some works have been revised and added to the paper. However, some clarifications are still needed:

1) The authors changed the claim “there are not may models available” concerning gamification for “there are some models available”. Again, authors do not clarify what they understand by “models” and, given that there are some models available, it would be advisable to cite them and discuss them.

Thank you for this comment. We had included information about models. We decided to modified this information with two new references:

(21) Hunicke R, Leblanc M, Zubek R, Mda: A formal approach to game design and game research. Proceedings of the AAAI Workshop on Challenges in Game AI, 2004;4:1-5.

(22) Osterwalder A, Pigneur Y. Business model generation: a handbook for visionaries, game changers, and challengers (Vol. 1). New Jersey: John Wiley & Sons, 2010.

2) The authors state the following “The usefulness of gamification in education have been proven in different context, because its implementation in the classrooms is still increasing [26, 27,28]. Until now, very few attempts have been made to apply gamification techniques to education Some teachers apply gamification in they own classes, for instance, discarding traditional grades in favour of experience points [29]”. First, it is claimed that the implementation in the classroom is increasing but then, it is stated that very few attempts have been made to apply gamification techniques, which, is confusing as it was previously said that gamification is increasing. In addition, again, this claim is inaccurate as there are plenty of works concerning gamification in education. This paragraph should be revised for coherence and consistency.

We also revised coherence and consistency. The final paragraph is:

The usefulness of gamification in education have been proven in different context~~, because~~. Nowadays, its implementation in the classrooms is still increasing [28, 29,30]. Until now, ~~very few attempts have been made to apply gamification techniques to education~~ some teachers apply gamification in they own classes, for instance, discarding traditional grades in favour of experience points [31].

Comments about the Introduction/discussion section

1) The authors have not clarified if the portrait given of digital native students (individualist, selfish, materialistic, etc.) is their own vision or not, as requested in previous revision. There is no clarification either on the revised version of the paper or in the response to reviewers.

Regarding of the portrait of digital native students as we indicate on the introduction is about the information of Prensky.

2) “The shortage of studies on the topic can explain why the students of Degree in Primary Education”. In my humble opinion, this claim is too risky. There are in fact many works on gamification, if the authors refer to some specific aspect of gamification that has not been sufficiently addressed in previous research, they should precise it. If they refer to gamification in general, this claim is not accurate.

We refer about the formation that the students of Degree in Primary Education have about gamification. But we prefer now to take it out of the text to avoid confusions.

3) This comment has not been addressed, either on the revised version of the paper or in the response to reviewers.

Definition of the term gamification. The authors state in the Discussion section “That term refers to the use of videogames on the school as a part of the teaching-learning process what it is supposed to motivate and get an engage with the pupils.” Why are the authors referring to videogames for defining gamification? In fact, this definition of gamification does not correspond to those offered by the authors in the Introduction section, in which they defined gamification as follows “Authors like Zichermann and Cunningham [12] define the term gamification as “the process of game-thinking and game mechanics to engage users and solve problems”. Others like Kapp[13] limit gamification to the use of mechanics, aesthetics and game thinking to attract people, motivate action, promote learning and solve problems. “No specific mention to videogames is made in these definitions. Definitions of gamification should be revised so they are coherent

We wrote this affirmation “That term refers to the use of videogames on the school as a part of the teaching-learning process what it is supposed to motivate and get an engage with the pupils” in the first version of the article. But in the July´s correction we changed it because we realized that it was our mistake. Also, in the last version we did not include it. Thus, if you need any explanation, we are glad to received it.

Comments about methodology

4) This comment has not been addressed, either on the revised version of the paper or in the response to reviewers.

Why do the authors use this reference “25) Sijtsma K. On the use, the misuse, and the very limited usefulness of Cronbach’s alpha. Psychometrika2009;74(1):107- 120. doi: 10.1007/S11336-008-9101-0” to justify that their questionnaire is reliable?. Sijtsma argues that Cronbach’s alpha suffers from major problems

Finally, thank you for this comment. We have decided to remove the reference because we had an interpretation error.
